# *In Vitro* Selection of Single-Stranded DNA Molecular Recognition Elements against *S. aureus* Alpha Toxin and Sensitive Detection in Human Serum

**DOI:** 10.3390/ijms16022794

**Published:** 2015-01-27

**Authors:** Ka L. Hong, Luisa Battistella, Alysia D. Salva, Ryan M. Williams, Letha J. Sooter

**Affiliations:** 1 Department of Pharmaceutical Sciences, West Virginia University, 1 Medical Center Drive, P.O. Box 9530, Morgantown, WV 26506, USA; E-Mails: khong@mix.wvu.edu (K.L.H.); lbattistella@mail.wvu.edu (L.B.); asalva@mix.wvu.edu (A.D.S.); rwilliams@mix.wvu.edu (R.M.W.); 2 Memorial Sloan Kettering Cancer Center, Molecular Pharmacology and Chemistry, 1275 York Avenue, P.O. Box 425, New York, NY 10065, USA

**Keywords:** SELEX, *in vitro* selection, aptamer, ssDNA, molecular recognition element (MRE), alpha toxin, *Staphylococcus aureus*, ELISA

## Abstract

Alpha toxin is one of the major virulence factors secreted by *Staphylococcus aureus*, a bacterium that is responsible for a wide variety of infections in both community and hospital settings. Due to the prevalence of *S. aureus* related infections and the emergence of methicillin-resistant *S. aureus*, rapid and accurate diagnosis of *S. aureus* infections is crucial in benefiting patient health outcomes. In this study, a rigorous Systematic Evolution of Ligands by Exponential Enrichment (SELEX) variant previously developed by our laboratory was utilized to select a single-stranded DNA molecular recognition element (MRE) targeting alpha toxin with high affinity and specificity. At the end of the 12-round selection, the selected MRE had an equilibrium dissociation constant (*K*_d_) of 93.7 ± 7.0 nM. Additionally, a modified sandwich enzyme-linked immunosorbent assay (ELISA) was developed by using the selected ssDNA MRE as the toxin-capturing element and a sensitive detection of 200 nM alpha toxin in undiluted human serum samples was achieved.

## 1. Introduction

Alpha toxin, also known as alpha-hemolysin, is a virulence factor secreted by *Staphylococcus aureus*, a facultative anaerobic Gram positive cocci bacteria [1]. *S. aureus* can cause a wide variety of infections in both healthy and hospitalized individuals, such as skin/soft tissue infections, bacteremia, pneumonia, and endocarditis [2]. Recent increases in the emergence of methicillin-resistant *S. aureus* (MRSA) in both health care settings and communities have raised global concerns [3,4]. It was estimated that there were approximately 300,000 patients in the US hospitalized with *S. aureus*-induced skin/soft tissues infections in 2007, with an average hospital stay of 4.5 days [5,6].

Most strains of *S. aureus* produce alpha toxin. It forms pores in target cell membranes, causing leakage of ions and cytolysis [1]. It has been shown that alpha toxin is involved in cell and tissue damage at infection sites and in inflammatory responses [7]. Antibodies against alpha toxin have been identified in patients with *S. aureus* infection, indicating the systemic involvement of alpha toxin in humans [8]. In addition, the important role of alpha toxin in pathogenesis has been reported in multiple previous studies [9,10,11].

Due to the problems associated with *S. aureus* infection, it is important to correctly diagnose these infections in a timely manner. The current diagnosis of *S. aureus* related infections are mostly designed for specific types of infections: echocardiography for patients with suspected *S. aureus* endocarditis and bacteria culturing from samples collected at sites of infections [12,13,14]. These methods are slow, non-specific and require multiple tests. Recently, PCR and Western blot/dot ELISA have been investigated to detect the presence of alpha toxin-coding genes and alpha toxin to facilitate the diagnosis of *S. aureus* related skin/soft tissue infections [15,16]. These methods are sensitive, but require laboratory equipment that may not be readily accessible in some hospitals. Other traditional ELISA assays have also been reported for alpha toxin detection [17,18]. However, the batch-to-batch variation in antibodies may hinder the standardization of these assays [19].

Single-stranded DNA molecular recognition elements (MRE) are an alternative to antibodies that have the potential to address the current limitations in diagnosing *S. aureus* infections. MREs can be proteins (antibodies or antibody fragments), small peptides or nucleic acids (aptamers or SOMAmers). They have high affinities and specificities toward the target of interest. The first nucleic acid MRE was described by the Gold laboratory in 1990, and was isolated using the Systematic Evolution of Ligands by Exponential Enrichment (SELEX) [20]. For single-stranded DNA (ssDNA) MREs, the process begins with incubating a large random library of different ssDNA molecules (10^13^ to 10^15^) with the target of interest. The library is then subject to repeated cycles of partitioning, amplification of bound library molecules, and removal of unbound molecules. One or a few MREs with high affinities and specificities toward the target of interest can be identified at the end of the *in vitro* selection process.

In this study, a rigorous SELEX scheme previously developed by our laboratory was used to identify a ssDNA MRE that binds to alpha toxin with high affinity and specificity [21,22,23]. The stringency of this SELEX variant is due to the focus on eliminating library binding to negative targets that are either structurally similar or likely to coexist in the same environment with the target of interest. These negative targets include bovine serum albumin, toxin B of *Clostridium difficile*, exotoxin A of *Pseudomonas aeruginosa* and cholera toxin of *Vibrio cholerae*. In addition, the identified alpha toxin-specific MRE has been utilized in a ssDNA MRE modified sandwich ELISA assay for the detection of the target in human serum samples at nanomolar concentrations.

## 2. Results and Discussion

### 2.1. Identification of Alpha Toxin Specific MRE

**Figure 1:**
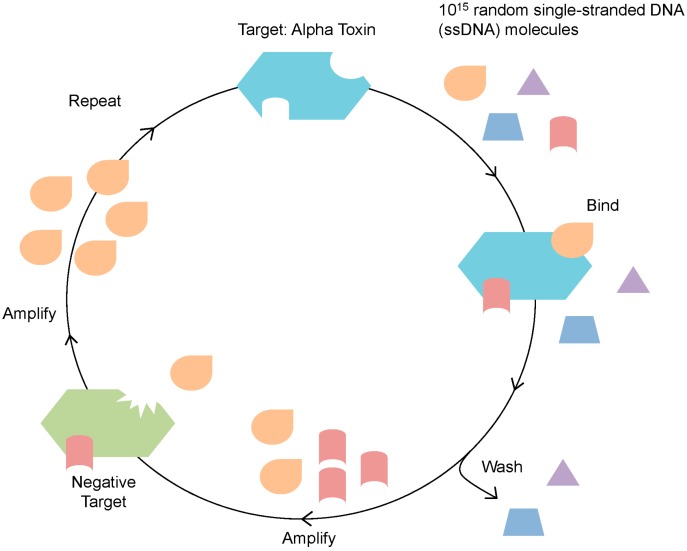
Illustration of the Systematic Evolution of Ligands by EXponential enrichment (SELEX) process. A random library consisting of 10^15^ ssDNA molecules (each with a different nucleotide sequence, indicated by different shapes) were incubated with the target alpha toxin. Those DNA that bound to the target were amplified and then incubated with negative targets. Those DNA that do not bind to negative targets are amplified and subjected to further rounds of *in vitro* selection.

**Figure 2:**
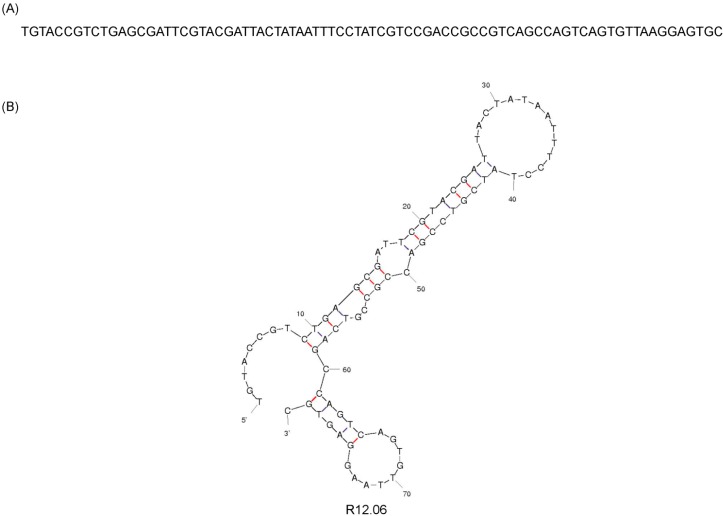
Sequence and secondary structure of R12.06 ssDNA MRE. Hydrogen bonds are indicated in red between GC base pairs and in black between AT base pairs. (**A**) ssDNA sequence of alpha toxin MRE R12.06; and (**B**) Mfold prediction of R12.06 secondary structure [24].

Twelve rounds of *in vitro* selection were performed to identify a ssDNA MREs against alpha toxin (Table 1, Figure 1). The selection utilized a SELEX scheme previously described by our lab [21]. This scheme was designed to enrich the ssDNA library to bind preferentially to alpha toxin in solution and to decrease binding to bovine serum albumin (BSA), toxin B, exotoxin A, and cholera toxin. Thirty to fifty random sequences were analyzed for the enrichment of consensus sequence families after every third round of selection (rounds 3, 6, 9, 12) to monitor the diversity of the library. The sequences from round 12 were analyzed for the presence of consensus sequences, but were also screened based on their predicted secondary structures and the stability of those structures, as predicted by a Gibbs free energy value. The random region of one sequence, R12.06 from the analyzed Round 12 library appeared to be highly conserved among several sequence families, and therefore it was chosen for further characterization (Table 2). The Mfold predicted secondary structure showed a long stem-loop structure comprised of the random region of the MRE and with a Gibbs free energy value of −8.85 kcal/mol (Figure 2). The entire random region of R12.06 participated in the formation of the long stem-loop secondary structure according to the Mfold prediction. The random region of R12.06 also shares approximately 30% and 50% identity with the random regions of R12.26 and R12.02 respectively.

**Table 1:** Systematic Evolution of Ligands by Exponential Enrichment (SELEX) scheme for ssDNA molecular recognition element (MRE) identification against alpha toxin.

Round	Positive Selection (+)	PCR Cycles	Negative Selection (–)	PCR Cycles
1	Immobilized Target (IT) 24 h	9	–	–
2	IT 18 h	15	BSA Immobilized Negative Target (INT) 22 h	16
3	IT 13 h	13	BSA INT 26 h	17
4	IT 7 h	18	Exotoxin A INT 22 h	16
5	IT 3 h	11	Exotoxin A INT 26 h	15
6	IT 30 min	17	BSA INT 24 h	12
7	IT 5 min, Competitive Elution with 1 mg/mL free alpha toxin, 5 min	17	IT 5 min, Competitive Elution with 1 mg/mL free BSA, 5 min	16
8	IT 5 s, Competitive Elution with 1 mg/mL free alpha toxin, 5 s	15	IT 5 s, Competitive Elution with 20 µg/mL free exotoxin a, 6 h	13
9	IT 5 s, Competitive Elution with 10 µg/mL free alpha toxin, 5 s	12	IT 5 s, Competitive Elution with 20 µg/mL free cholera toxin, 6 h	13
10	IT 5 s, Competitive Elution with 5 µg/mL free alpha toxin, 5 s	12	IT 5 s, Competitive Elution with 20 µg/mL free toxin B, 6 h	13
11	IT 5 s, Competitive Elution with 2.5 µg/mL free alpha toxin, 5 s	19	IT 5 s, Competitive Elution with 20 µg/mL free BSA, 24 h	7
12	IT 5 s, Competitive Elution with 1 µg/mL free alpha toxin, 5 s	10	–	–

*In vitro* selection performed for identifying alpha toxin specific ssDNA MRE. Immobilized target (IT) is alpha toxin bound to magnetic beads. Immobilized negative target (INT) are negative targets bound to magnetic beads. BSA is the abbreviation for bovine serum albumin. Times listed are incubation times in hours (h), minutes (min) or seconds (s).

**Table 2:** Sequence families after 12 rounds of SELEX.

Designation Sequence
R12.26 TGTACCGTCTGAGCGATTCGTACCCTTGC CGATGC$\underline{\underline{\text{CT}}}$T$\underline{\underline{\text{TA}}}$CGG$\underline{\underline{\text{TC}}}$TAG$\underline{\underline{\text{T}}}$TTGGATGTAGCCAGTCAGTGTTAAGGAGTGC
R12.06 TGTACCGTCTGAGCGATTCGTACGATTA$\underline{\underline{\text{CT}}}$A$\underline{\underline{\text{TA}}}$ATT$\underline{\underline{\text{TC}}}$CTA$\underline{\underline{\text{T}}}$CGTCCGACCGCCGTCAGCCAGTCAGTGTTAAGGAGTGC
R12.01 TGTACCGTCTGAGCGATTCGTACTCGGGCGATGATACTTAGCACGGTCTAGGTCAAAAGCCAGTCAGTGTTAAGGAGTGC
R12.20 TGTACCGTCTGAGCGATTCGTACTAGCGGCAGAGTAGCACTCTATAGGTCGATGTTTAGCCAGTCAGTGTTAAGGAGTGC
R12.01 TGTACCGTCTGAGCGATTCGTACTCGGGCGATGATACTTAGCACGGTCTAG GTCAAAAGCCAGTCAGTGTTAAGGAGTGC
R12.02 TGTACCGTCTGAGCGATTCGTACCG$\underline{\underline{\text{T}}}$GTCC$\underline{\underline{\text{TA}}}$T$\underline{\underline{\text{TTTCTT}}}$C$\underline{\underline{\text{TCT}}}$GTTA$\underline{\underline{\text{AC}}}$TCTCGTCAGCCAGTCAGTGTTAAGGAGTGC
R12.06 TGTACCGTCTGAGCGATTCGTACGAT$\underline{\underline{\text{T}}}$ACTA$\underline{\underline{\text{TA}}}$A$\underline{\underline{\text{TTTCTT}}}$A$\underline{\underline{\text{TC}}}$GTCCG$\underline{\underline{\text{AC}}}$CGCCGTCAGCCAGTCAGTGTTAAGGAGTGC
R12.39 TGTACCGTCTGAGCGATTCGTACTTTGATCTCGTGTGTCTAGTTGCGGCGGATTGTCAGCCAGTCAGTGTTAAGGAGTGC
R12.10 TGTACCGTCTGAGCGATTCGTACGGTCAACCTCACCGACTGCCGACCGTTTAATTCGAGCCAGTCAGTGTTAAGGAGTGC
R12.43 TGTACCGTCTGAGCGATTCGTACCGTCATTGCCTCGTAGTATTCTTATAGTCGGTAGAGCCAGTCAGTGTTAAGGAGTGC
R12.44 TGTACCGTCTGAGCGATTCGTACTCCCGAAAGCGCGTCAGCCTGGGAGGTTATGCGGAGCCAGTCAGTGTTAAGGAGTGC

Representative sequence families from Round 12 ssDNA library. Families are separated according to their consensus sequences (underline) and sequence homologies (double-underline).

### 2.2. Affinity and Specificity of Alpha Toxin Specific MRE

Surface plasmon resonance single cycle kinetics assays were used to determine the affinity of R12.06 for alpha toxin. The average equilibrium dissociation constant (*K*_d_) was 93.7 ± 7.0 nM (Table 3). Single cycle kinetics have previously been used to determine the binding affinities of nucleic acid MREs [25,26]. This assay is typically used for sensor surfaces that are difficult to regenerate and cannot therefore undergo classical multi-cycle kinetic analysis. Single cycle kinetics has also been shown to provide equally valid results as multi-cycle assays [27,28]. Ligand (ssDNA MRE) immobilization strategies described in this work differ from many previous studies [25,29,30,31]. Here, covalent linkage of 5'-amino modified ssDNA MRE was performed instead of the more typical biotin-streptavidin capturing. At neutral to slightly basic running buffer, the electrical repulsion between the negatively charged DNA and sensor chip surface leads to relatively lower level of ligand immobilization by covalent attachment. Both sensor chips produced in house and commercial CM5 sensor chips were utilized in the assays. A different concentration range (25 to 500 nM) of alpha toxin was also tested and yielded a comparable result (*K*_d_ = 90.7 nM), and thus confirming the validity of the determined equilibrium dissociation constant. The *K*_d_ of R12.06 is comparable to other previously reported *K*_d_ values of MREs targeting bacterial toxins [32,33,34]. This further validates the SELEX variation previously developed by our laboratory [21,22,23].

**Table 3:** Surface plasmon resonance (SPR) affinity data of R12.06 ssDNA MRE.

Assay	*K*_d_ (nM)	χ^2^ (RU)^2^
Assay 1	102	0.493
Assay 2	88.7	0.691
Assay 3	90.7	0.164
Averaged	93.7 ± 7.0	–

The averaged equilibrium dissociation constant is given with standard deviation from three assays. The χ^2^ described the closeness of fit between the experimental and fitted curve. RU represents the response unit generated by the SPR instrument.

A fluorescent plate-based assay was used to determine the cross binding activity of R12.06. This assay was slightly modified from that which is previously described by using a different washing buffer [33]. The data is presented relative to binding between R12.06 and alpha toxin as has been previously described [21,22]. The ssDNA MRE exhibits significant binding preference to alpha toxin over all negative targets (*p* < 0.05) (Table 4). The binding of R12.06 to alpha toxin is 1.5 times greater than cholera toxin and exotoxin A (*p* = 0.003 and *p* = 0.004 respectively), 5.0 times greater than toxin B (*p* = 0.0005), and 1.8 times greater than BSA (*p* = 0.03). It is important to note that components of human serum were not included in the negative selection scheme, but R12.06 still shows 1.7 times greater selectivity over human serum (*p* = 0.02). This selectivity is important for downstream application development. An interesting phenomenon observed in this study is that the binding selectivity over toxin B (270 kDa) is more than triple that of cholera toxin (84 kDa). However, both toxins were only introduced once in the negative selection scheme. A similar phenomenon was also observed in another study from the Sooter laboratory that investigated the binding selectivity of a *C. difficile* toxin B specific ssDNA MRE over other toxins, in which the toxin B MRE is two times more selective of alpha toxin (33 kDa) over cholera toxin. Based upon these two observations, it is likely that the binding selectivity of the ssDNA library is enriched early on in the selection process and the target molecular weight and crystal structure may play a role in the selectivity of MREs. The overall low cross binding activities in all tested negative targets further validates the stringency of our selection process [21,22,23].

**Table 4:** Cross-reactivity data of R12.06 ssDNA MRE.

Target	Normalized Average Fluorescence	Standard Deviation	*p*-Value	Selective Ratio
Alpha Toxin	0.047	0.007	–	–
Cholera Toxin	0.031	0.009	0.003	1.5
Exotoxin A	0.031	0.002	0.004	1.5
Toxin B	0.009	0.002	0.001	5.0
Bovine Serum Albumin	0.026	0.001	0.027	1.8
Human Serum	0.028	0.003	0.017	1.7

For each protein target, normalized average fluorescence is given with 1× standard deviation. The *p*-value is calculated from a (one tailed) student’s *t*-test between alpha toxin and cross-binding targets. The selectivity ratio represents the number of times greater binding to alpha toxin than to other cross-binding targets.

### 2.3. Diagnostic Application of Alpha Toxin Specific MRE

**Figure 3:**
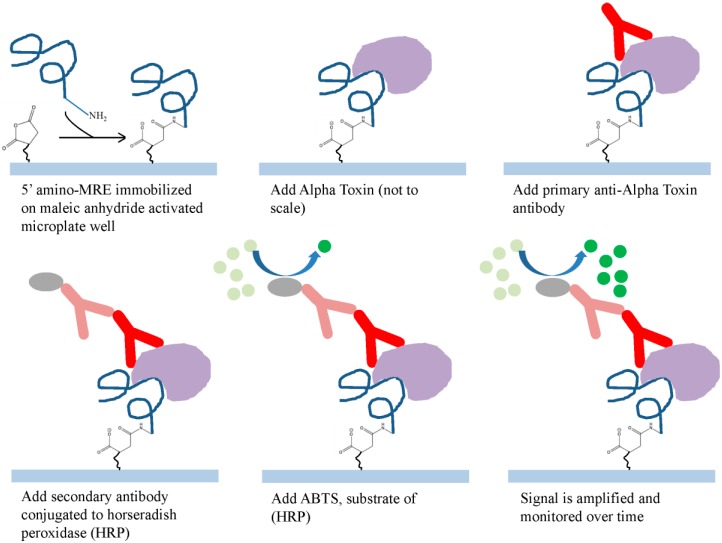
Illustration of the ssDNA MRE modified ELISA assay. The ssDNA MRE was used as the capturing element in the sandwich ELISA assay. The curved blue line attached to NH_2_ represents the 5' amino modified MRE. The purple irregular shape represents alpha toxin. The red “Y” shape represents the primary antibody against alpha toxin. The grey oval represents horseradish peroxidase (HRP) conjugated to a secondary antibody (pink). The light green circle represents the substrate of HRP and the dark green circle represents the metabolite of HRP, which is detected with absorbance measurements.

**Figure 4:**
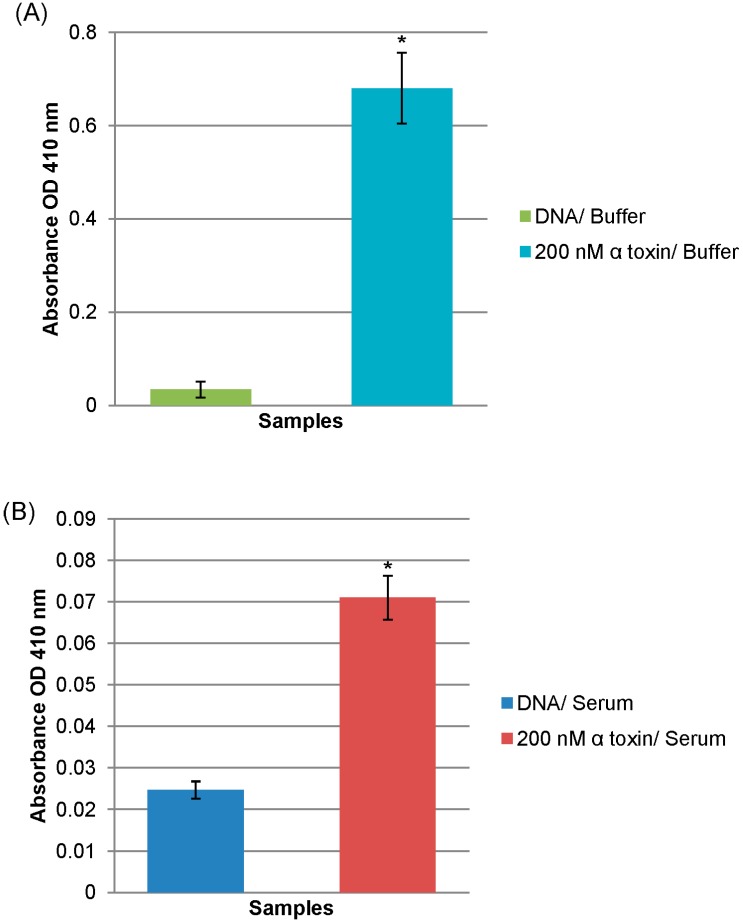
Detection of alpha toxin in modified ELISA assay. Data represent one modified sandwich ELISA with absorbance measured at 410 nm. Absorbance levels are subtracted from background levels of blank wells without immobilized DNA. (**A**) Statistical significance levels with respect to DNA with buffer background of *p* < 0.001 are designated by *; and (**B**) Statistical significance levels with respect to human serum background of *p* < 0.001 are designated by *. Buffer: 1× selection buffer. Error bars are representative of ±1× standard deviations.

The high affinity and specificity of the alpha toxin specific ssDNA MRE allowed the investigation of its potential application as a diagnostic tool. A modified ELISA using R12.06 as the toxin-capturing element was developed (Figure 3). Reproducible and statistically significant detection of 200 nM alpha toxin spiked in human serum samples were achieved compared to control in multiple assays (*p* < 0.01 to *p* < 0.001) (Figure 4). The significant differences were first detected 20 min after the addition of horse radish peroxidase substrates. It is important to note that the average half-life of ssDNA MREs in serum is about one hour due to the presence of exonucleases [35]. The R12.06 MRE demonstrated its robustness in serum without any base modifications. The three dimensional structure of nucleic acid MREs are known to be influenced by temperature, pH and ionic strength of the binding conditions [36]. The R12.06 MRE was able to retain a level of affinity and specificity in undiluted human serum, which is a complex biological matrix with serum proteins, lipids and varied ionic concentrations [37]. The assay completion time from the addition of alpha toxin to positive result was less than 4 h. This demonstrates the potential of R12.06 as a clinical diagnostic tool.

A single-stranded DNA MRE was previously incorporated into a system for target detection in binding buffer-diluted human serum [38]. Similar ssDNA MRE based ELISA assays have also been reported in previous studies for the detection of bacterial toxin targets in binding buffer [33,39]. In comparison, the assay in this study demonstrates a level of superiority by detecting target molecules in minimally manipulated and clinically relevant samples. Traditional antibody-based ELISA assays for the detection of alpha toxin have been previously reported with high sensitivity in bacterial culture media (LOD of 1 ng/mL) [17,18]. In these experiments 200 nM, or 6.6 µg/mL, alpha toxin in human serum was detected. While highly reproducible, the signal is small enough to be near the limit of detection for the system described. This level is within the range of clinical relevance, as previous work has shown levels to be as high as 83 µg/mL [40]. The sensitivity of the assay may be improved by making the ssDNA MRE more resistant to exonucleases. This stability limitation may be resolved by base modifications [41]. In contrast to antibodies, ssDNA MREs also have several advantages, such as thermostability, reversible denaturation and inexpensive chemical synthesis [42]. It is to be noted that another ssDNA MRE targeting alpha toxin has been reported recently [43]. The authors investigated the potential therapeutic application of their selected MRE, however, no binding affinity and specificity data were reported in the study. It is unknown if R12.06 will demonstrate neutralizing effect on alpha toxin. Based upon the determined high affinity and specificity of R12.06, its translational value may not be limited to diagnostic detection and may warrant future studies. In sum, the ability of R12.06 to detect alpha toxin in undiluted human serum samples has been demonstrated, which has the potential to augment current and future diagnostic methods for *S. aureus* related infections.

## 3. Experimental Section

### 3.1. SELEX for Identification of Alpha Toxin Specific MREs

**Figure 5:**
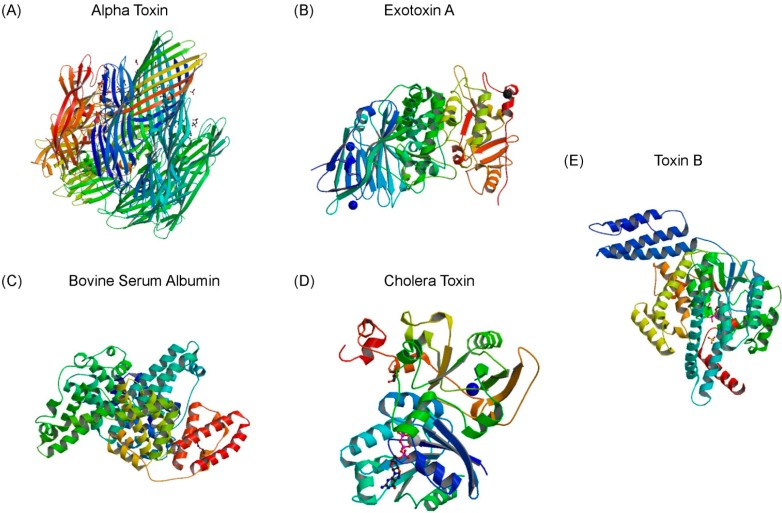
Structures of targets used in the SELEX scheme and cross binding assays. (**A**) Ribbon structure of the target of interest, alpha toxin (PDB 3ANZ, 33 kDa) [44]; (**B**–**E**) Ribbon structure of exotoxin A (PDB 1IKQ, 66 kDa) [45], bovine serum albumin (PDB 4F5S, 66.5 kDa) [46], cholera toxin (PDB 2A5D, 84 kDa) [47], and toxin B (PDB2BVM, 270 kDa) [48], used in negative rounds of selection and cross binding assays.

The *in vitro* selection process started from a single-stranded DNA (ssDNA) library consisting of 10^15^ different molecules (Figure 1). This library was previously designed by our laboratory, termed RMW.N34 [21]. It consisted of two 23 base constant regions for polymerase chain reaction (PCR) amplification flanking by a 34 base random region (synthesized by Eurofins MWG Operon, Huntsville, AL, USA). Twelve rounds of SELEX were carried out to select ssDNA molecules that bound to alpha toxin and those that bound to negative targets were eliminated in the process (Table 1, Figure 5).

Lyophilized alpha toxin (List Biological Laboratories, Campbell, CA, USA) was reconstituted in pure water and conjugated to carboxylic acid-coated magnetic beads (Dynabeads M-270 Carboxylic Acid, Life Technologies, Grand Island, NY, USA) via a two-step amidation reaction using *N*-hydroxysulfonyl succinimide (sulfo-NHS) (Pierce, Rockford, IL, USA) and 1-ethyl-3-(3-dimethylaminopropyl) (EDC) (Pierce). The reaction was performed according to manufacturer’s protocol.

For positive rounds of immobilized target selection, the ssDNA library was incubated with 6 µL of immobilized target in 200 µL of selection buffer (100 mM sodium chloride, 20 mM Tris-HCl, and 2 mM magnesium chloride; 1× selection buffer, SB) at room temperature with rotation. After incubation, the solution was then subjected to magnetic separation. Unbound ssDNA in solution was removed and immobilized target with bound ssDNA was washed three times with 200 µL of SB and resuspended in 100 µL of SB. This suspension served as template for PCR amplification. The PCR conditions were as follows: bound ssDNA, 400 nM forward and biotinylated reverse RMW.N34 primers (Eurofins MWG Operon) (forward primer sequence: 5'-TGTACCGTCTGAGCGATTCGTAC-3', biotinylated reverse primer sequence: 5'-Biotin-GCACTCCTTAACACTGACTGGCT-3'), 250 µM deoxynucleotide triphosphates, 1× GoTaq Reaction Buffer (Promega, Madison, WI, USA), 3.5 units *Taq*, and pure water. Thermal cycling conditions were as follows: denature at 95 °C for 5 min, cycle at 95 °C for 1 min, 63 °C for 45 s, and 72 °C for 1 min; and final extension temperature at 72 °C for 7 min [21,22,23]. Large-scale 3 mL PCR was performed after each round of positive and negative selection. This selection process for immobilized alpha toxin target was carried out for Rounds 1–6, each with shortened incubation periods.

Amplified PCR product containing dsDNA was purified with the IBI PCR purification kit (IBI Scientific, Peosta, IA, USA) according to manufacturer’s protocol. Single strand separation and ethanol precipitation of the forward strand DNA were performed identically to as previously described [21,22,23]. This procedure was carried out after each round of positive and negative selection.

For negative rounds of selection, BSA and Exotoxin A (List Biological Laboratories) were conjugated to carboxylic acid-coated magnetic beads as described above and served as immobilized negative targets. Selection procedures were carried out similarly to positive Rounds 1–6. However, unbound ssDNA in solution served as template for PCR amplification. This procedure was performed for negative Rounds 2–6.

Free alpha toxin in solution was used to perform competitive elutions beginning in Round 7 positive. The ssDNA library was first incubated with immobilized target as described for positive Round 1–6. However, free alpha toxin at a concentration of 1 mg/mL in SB was used to resuspend the ssDNA bound magnetic beads. The solution containing ssDNA bound to free alpha toxin was retrieved by magnetic separation after 5 min of incubation and served as template for PCR amplification. This procedure was carried out for positive Rounds 7–12, each with shorter incubation times and lower free alpha toxin concentrations. Similar competitive elution with free negative targets in SB was performed for negative Rounds 7–11. However, beads were retrieved and resuspended in 100 µL of SB and served as template for PCR amplification.

### 3.2. Cloning and Sequencing of Alpha Toxin Specific MREs

DNA sequencing was performed following Rounds 3 negative, 6 negative, 9 negative and 12 positive to analyze the enrichment of consensus binding sequences in the ssDNA library. This was performed identically to as previously described [21,22,23]. A total of thirty to fifty randomly selected sequences were analyzed for each sequenced round.

### 3.3. Alpha Toxin MRE SPR Affinity Binding Assays

One candidate sequence designated as R12.06 from the analyzed round 12 library was chosen for further characterization. Mfold DNA web server was used to predict the secondary structure of R12.06 with parameter settings at the ionic conditions of SB and at 25 °C [24]. R12.06 was commercially synthesized with a 5' amino-C6 modification for the use of surface plasmon resonance (SPR) affinity binding assays (Eurofins MWG Operon). The 5' end was chosen for the amino modification because in the Mfold predicted secondary structure (Figure 2) the 5' end was further away from predicted secondary structures than the 3' end. This provided distance between the immobilization surface and the MRE structure in addition to the C6 linker between the DNA and the amino group. A commercially-purchased CM5 SPR sensor chip (GE Healthcare, Piscataway, NJ, USA) and a sensor chip fabricated in house were used in the assays.

Glass slides (12 mm × 10 mm) were coated with a 2 nm titanium adhesion layer and a 45 nm gold layer using Temescal BJD-2000 system (Edwards Vacuum, Phoenix, AZ, USA) with an Inficon XTC/2 deposition controller (Infincon, East Syracuse, NY, USA). In order to assemble the home-made sensor chip, the gold coated glass slide was first washed with 100% ethanol under sonication for 5 min, then immersed in a self-assembled monolayer solution of 10 mM 11-mercaptoundecanoic acid (11-MUA) (Sigma, St. Louis, MO, USA) and 10 mM triethylene glycol mono-11-mercaptoundecylether (PEG3) (Sigma) in a 1 to 5 ratio overnight under argon. After overnight incubation in the solution, the gold chip was rinsed with 100% ethanol and pure water, blown dry with nitrogen and assembled onto a carrying cartridge. A Biacore X100 (GE Healthcare) was used for binding assays.

Both types of sensor chips were first activated by injecting 100 mM *N*-hydroxysulfonyl succinimide (sulfo-NHS) (Pierce) and 400 mM 1-ethyl-3-(3-dimethylaminopropyl) (EDC) (Pierce) at a 1 to 1 ratio to control and active flow cells at a flow rate of 5 µL/min for ten minutes. Then, 5' amino-C6 modified R12.06 was diluted to 100 nM in immobilization buffer (100 mM sodium chloride, 20 mM potassium phosphate, and 2 mM magnesium chloride, pH 7.4). This buffer was also used as the running buffer for the DNA immobilization step. A total of 300 µL of DNA was injected into active flow cell at a flow rate of 5 µL/min, followed by a 10-min inactivation step using 1 M ethanolamine-HCl pH 8.5. Control flow cell without immobilized DNA was also inactivated by 1 M ethanolomine-HCL.

The selection buffer was then used as running buffer for single cycle kinetic assays. Alpha toxin at various concentrations (500, 750, 1000, 1500, 2000 and 2500 nM) in SB were injected into both control and active flow cells at a flow rate of 30 µL/min for 180 s with a dissociation time of 150 s. Binding responses after baseline and control adjustments were analyzed with Biacore X100 evaluation software (GE Healthcare). A 1:1 binding model was used to determine the equilibrium dissociation constant (*K*_d_).

### 3.4. Alpha Toxin MRE Fluorescence cross Binding Assays

Commercially synthesized FAM labeled R12.06 (Eurofins MWG Operon) was used in microplate based fluorescence cross binding assays. The assay was slightly modified from a previous study [33]. Alpha toxin, exotoxin A, toxin B (List Biological Laboratories), cholera toxin (List Biological Laboratories) and BSA were diluted to 40 nM in 50 mM carbonate/bicarbonate buffer (pH 9.6). A volume of 90 µL of each diluted toxin, human serum (Sigma) and control blocking buffer (SB with 0.05% Tween-20) were added to individual wells of a 96 well Nunc C8 Lockwell MaxiSorp microplate (Pierce). The plate was incubated on a shaker at 4 °C overnight. After protein coating, all the wells were blocked for 1 h and washed 3 times with the blocking buffer at room temperature. FAM labeled R12.06 at 100 nM in 90 µL of SB was added to control and cross binding target coated wells and incubated for 1 h at room temperature. Subsequently, unbound FAM-R12.06 was removed and each well was washed 5 times with selection buffer. Lastly, 90 µL of SB was added to each well and the plate was measured in a Synergy 2 microplate reader with excitation at 490 nm and emission at 520 nm (Biotek US, Winooski, VT, USA). Fluorescence measurements were normalized to control and an internal standard of 90 µL of 100 nM FAM-R12.06 in SB as previously described [21,22,23]. All cross binding targets and control well sets were in triplicate. Data was averaged and standard deviation was calculated. A one-tailed student *t*-test was used to determine the statistical significance in difference of the means (*p* < 0.05).

### 3.5. Alpha Toxin MRE Modified ELISA

A 100 µL sample of commercially synthesized 5' amino-C6 modified R12.06 was diluted to 40 nM in immobilization buffer and added to individual wells of a maleic anhydride activated microplate (Pierce) and incubated overnight with shaking at room temperature. Each well was then blocked with blocking buffer (SB, 0.1% BSA and 0.05% Tween-20) for 1 h and washed 3 times with SB/0.05% Tween-20 washing buffer at room temperature. Wells without DNA added served as blank control. SB, human serum, and alpha toxin diluted to 200 nM in SB and in human serum were added into individual wells and incubated on a shaker for 1 h at room temperature. SB and human serum served as background control.

After incubation, all of the contents were aspirated and wells were washed 5 times with the same washing buffer. Then, 100 µL of rabbit anti-alpha toxin primary antibody serum (Sigma, St. Louis, MO, USA) at 1 to 500 dilution ratios in washing buffer was added and incubated for 30 min with shaking at room temperature. Primary antibody was then aspirated and wells were washed for 3 times. A secondary goat anti-rabbit antibody conjugated to horseradish peroxidase (Pierce) at 1 to 500 dilution ratios in washing buffer was added and incubated for 30 min with shaking at room temperature. Lastly, all contents were aspirated and washed 5 times as outlined above. ABTS substrate (Pierce) was then added to all wells according to manufacturer’s instruction. Absorbance at 410 and 650 nm was measured in a Synergy 2 microplate reader using Gen 5 1.06 software (Biotek US, Winooski, VT, USA) in two minute increments. Negative controls were wells incubated without antibodies and with only primary antibody. Each set was performed in triplicate. Data was averaged and standard deviations were determined. A two-tailed student *t*-test was performed to determined statistical differences at *p* < 0.05.

## 4. Conclusions

This study utilized a robust SELEX methodology to identify a molecular recognition element specific for alpha toxin of *Staphylococcus aureus* with high affinity and specificity. The MRE binds with a nanomolar equilibrium dissociation constant and is selective for alpha toxin over all bacteria toxins used in the negative selection scheme and in human serum. In addition, a proof-of-concept diagnostic sandwich ELISA utilizing the MRE as the toxin capturing element has been developed and successfully demonstrated target detection in clinically relevant samples. The results further validate our SELEX process and showed the potential of applying ssDNA MREs in diagnostic applications.
